# Comparative Characterization of σ^32^-Dependent Promoters for the Heat-Inducible Expression of FAST-PETase in *Escherichia coli*

**DOI:** 10.3390/ijms27177762

**Published:** 2026-08-30

**Authors:** Praopim Limsakul, Natcha Rasitanon, Sahban Da-oh, Amornrat Phongdara, Aekkaraj Nualla-ong, Krit Charupanit

**Affiliations:** 1 Division of Physical Science, Faculty of Science, Prince of Songkla University, Songkhla 90110, Thailand; praopim.l@psu.ac.th; 2 Center of Excellence for Trace Analysis and Biosensor (TAB-CoE), Prince of Songkla University, Songkhla 90110, Thailand; 3 Department of Biomedical Sciences and Biomedical Engineering, Faculty of Medicine, Prince of Songkla University, Songkhla 90110, Thailand; 6810330024@psu.ac.th; 4 Division of Biological Science, Faculty of Science, Prince of Songkla University, Songkhla 90110, Thailand; sahban0774@gmail.com; 5 Center for Genomics and Bioinformatics Research, Faculty of Science, Prince of Songkla University, Songkhla 90110, Thailand; pamornra@yahoo.com

**Keywords:** FAST-PETase, PET-degrading enzyme, heat-inducible expression, σ^32^-dependent promoter, heat shock response

## Abstract

Efficient regulation of recombinant enzyme expression is an important consideration for the development of microbial biocatalysts. Heat-inducible promoters regulated by the alternative sigma factor σ^32^ provide an inducer-free strategy for controlling gene expression in *Escherichia coli*. In this study, four σ^32^-dependent promoters (P*dnaK*, P*grpE*, P*ibpA*, and P*clpB*) were comparatively characterized using the PET-degrading enzyme FAST-PETase fused to superfolder green fluorescent protein as a model recombinant protein. Promoter performance was evaluated based on basal leakage, induction kinetics, and expression strength following heat induction. Among the promoters examined, P*dnaK* exhibited the strongest heat-inducible expression and was dissected to examine the autonomous and combinatorial behavior of its promoter-derived elements. Molecular docking analysis further supported the experimental observations by showing qualitative agreement between predicted σ^32^–DNA interactions and promoter performance. Together, these findings provide a comparative characterization of σ^32^-dependent promoters and identify promoter architectures that may facilitate the development of heat-inducible recombinant enzyme expression systems in *E. coli*.

## 1. Introduction

Polyethylene terephthalate (PET) is one of the most widely used plastics due to its chemical stability and mechanical durability, which make it suitable for a broad range of industrial applications. These properties contribute to significant environmental concerns, as PET is poorly biodegradable and accumulates in ecosystems [1,2,3]. Several studies have reported the presence of PET-derived microplastics in the human food chain and in various human biological samples [4,5,6,7], although their effects on human health are still under investigation. These observations underscore the urgent need for effective strategies to reduce PET accumulation. Enzymatic degradation has emerged as a sustainable strategy for PET recycling [8,9,10]. Numerous protein engineering efforts have improved the catalytic efficiency and thermostability of PET hydrolases [11,12,13]. Among the engineered variants, FAST-PETase exhibits enhanced degradation performance under mild conditions, making it a promising candidate for enzymatic PET recycling [14]. In addition to improving catalytic performance, considerable effort has focused on improving PETase production. To improve solubility and secretion, strain selection, disulfide bond-promoting expression hosts, and signal peptide engineering have been explored [15,16,17]. Secretion platforms in *Bacillus subtilis* and surface display systems in *Saccharomyces cerevisiae* have been designed to avoid the inclusion body formation frequently observed during cytoplasmic expression [18,19]. Highly soluble fusion tags (e.g., NusA or MBP), cytoplasmic chaperone co-expression (GroEL/ES), and periplasmic targeting signal peptides have also been shown to improve PETase yields [15,20,21].

Recombinant protein production in *Escherichia coli* remains one of the most widely used platforms for research and industrial biotechnology because of its rapid growth, well-established genetic tools, and cost-effective cultivation. Most recombinant proteins are produced using the T7 expression system induced by isopropyl β-D-1-thiogalactopyranoside (IPTG), which provides high transcriptional output and robust protein production. However, IPTG-based systems often exhibit leaky basal expression [22,23], and can impose a metabolic burden on host cells through competition for cellular resources [24,25] that could affect production efficiency. Techno-economic analyses have further estimated that IPTG alone can account for approximately 10–28% of total production costs depending on production scale and context [26,27]. Consequently, alternative induction strategies that avoid chemical inducers continue to be explored for recombinant protein expression.

Heat-inducible expression systems offer an alternative because gene expression can be triggered by a temperature shift without additional chemical inputs [28,29]. In *E. coli*, the heat shock response is primarily regulated by the alternative sigma factor σ^32^ (encoded by *rpoH*), which redirects RNA polymerase to specific heat shock (HS) promoters following an increase in temperature [30,31]. Under non-heat-shock conditions, intracellular σ^32^ levels remain low due to the negative regulation by the DnaK and GroE chaperone systems [30,31,32]. In particular, the DnaK system inhibits σ^32^ by preventing its association with RNA polymerase and facilitating its degradation via the FtsH protease [33,34]. Upon heat stress, however, σ^32^ rapidly accumulates and activates transcription of heat shock genes involved in protein folding and stress adaptation [31,32]. This natural regulatory network provides a framework for developing heat-inducible recombinant protein expression systems that link target gene expression with the endogenous cellular stress response [28]. Accordingly, σ^32^-dependent promoters have been applied in synthetic biology applications to control recombinant protein expression [28]. For example, HS promoters such as P*dnaK* and P*ibpA* have been used to construct whole-cell biosensors that convert stress responses into fluorescent, luminescent, or electrochemical signals [35,36]. Furthermore, co-expression of heat shock chaperones has been shown to improve the folding and solubility of recombinant proteins [37,38].

Although a recent study showed that co-expressing DnaK/DnaJ chaperones improved soluble FAST-PETase yields [15], this approach still relied on conventional IPTG-inducible expression systems. Here, four *E. coli* σ^32^-dependent promoters (P*dnaK*, P*grpE*, P*ibpA*, and P*clpB*) were systematically characterized for heat-inducible recombinant protein expression using FAST-PETase fused to superfolder green fluorescent protein (sfGFP) as a reporter for real-time monitoring of expression. Promoter performance was evaluated by comparing basal leakage, induction kinetics, and expression levels following heat induction. Since P*dnaK* showed the highest performance, its promoter-derived elements were further examined to assess their autonomous and combinatorial contributions to promoter activity. Molecular docking analysis was also performed to offer structural context for the observed differences among the individual P*dnaK* promoter-derived elements. These findings provide a systematic characterization of σ^32^-dependent promoter behavior and offer guidance for engineering heat-inducible promoters for recombinant protein expression in *E. coli*.

## 2. Results

### 2.1. Validation of the FAST-PETase-sfGFP Reporter System

Prior to the characterization of the regulatory properties of σ^32^-dependent promoters, the catalytic activity of codon-optimized FAST-PETase was verified using purified enzyme. PET degradation was confirmed by FTIR spectroscopy, weight-loss analysis, and SEM (Figure S1). These results confirmed that the codon-optimized FAST-PETase retained catalytic activity and was suitable for use as the recombinant protein model in this study.

**Figure 1:**
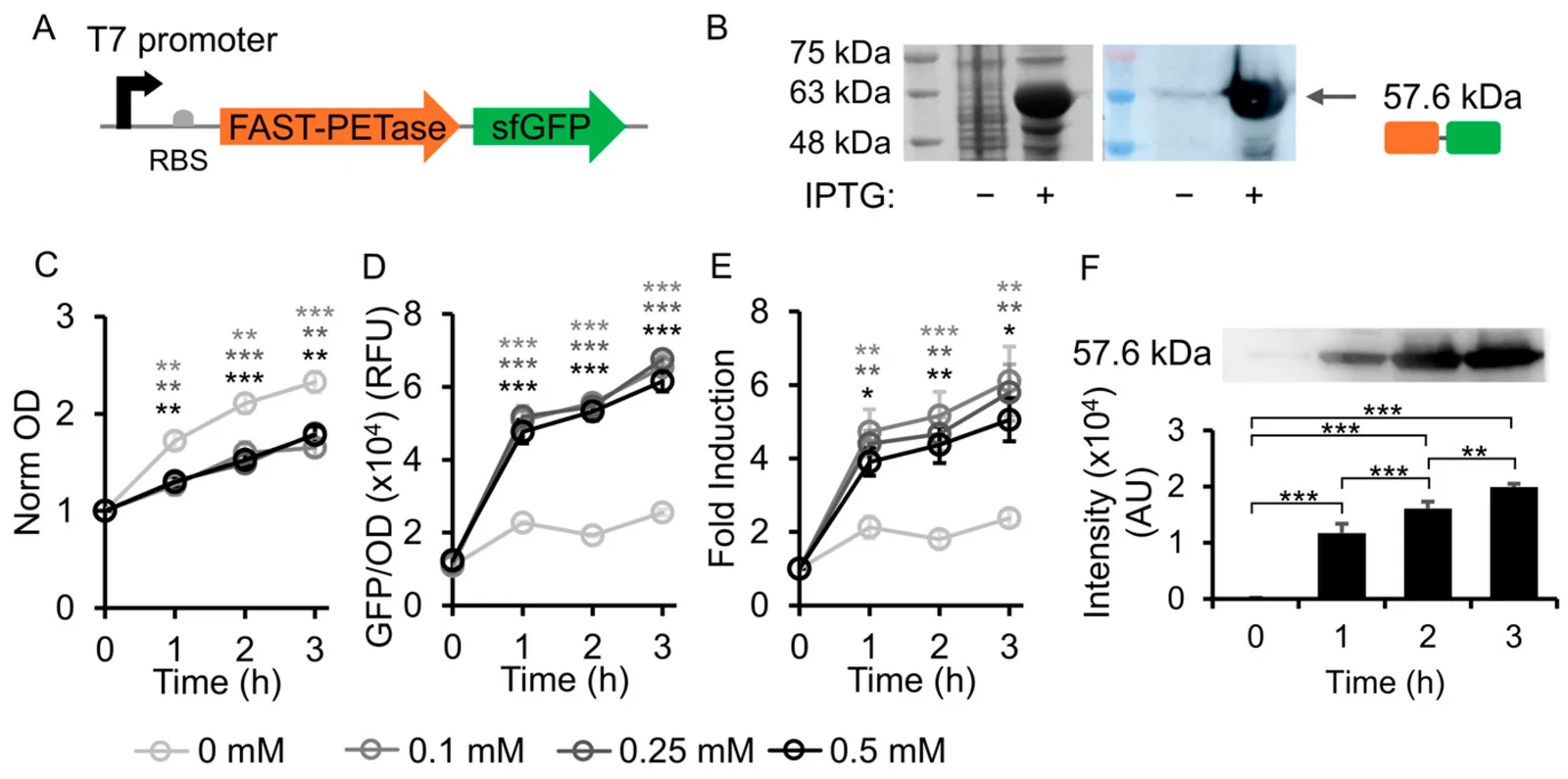
Reporter system for FAST-PETase expression. (**A**) Schematic of FAST-PETase–sfGFP constructs driven by the T7 promoter. (**B**) SDS-PAGE (left) and Western blot (right) analysis of FAST-PETase-sfGFP expression under non-induced (− IPTG) and induced (+ IPTG) conditions. The expected molecular weight of FAST-PETase-sfGFP is 57.6 kDa. Time-course expression of FAST-PETase-sfGFP was evaluated by (**C**) OD_600_ normalized to the pre-induction (0 h) value, (**D**) GFP fluorescence normalized to OD_600_ (GFP/OD, RFU), shown as absolute values to capture differences in expression magnitude across IPTG concentrations, and (**E**) the same data as in (**D**), normalized to the pre-induction (0 h) value for each condition and expressed as fold induction relative to basal expression. (**F**) Western blot analysis of samples collected at 0–3 h post-induction, with corresponding quantification of band intensity. Data in (**C**–**F**) are presented as mean ± SD, *n* = 3 independent biological replicates. Statistical significance was assessed by one-way ANOVA followed by Tukey’s HSD post hoc test. Asterisks indicate significant pairwise differences (* *p* < 0.05, ** *p* < 0.01, and *** *p* < 0.001). For clarity (**C**–**E**), only comparisons between each IPTG concentration and the non-induced (0 mM IPTG) condition at the corresponding time point are shown.

To enable real time monitoring of protein expression during promoter characterization, a fluorescence reporter system was developed. FAST-PETase was fused at its C-terminus to sfGFP (Figure 1A). Expression of the FAST-PETase–sfGFP fusion protein under the IPTG-inducible T7 promoter was validated by SDS-PAGE and Western blot analysis, which showed the expected 57.6 kDa fusion protein after induction, while no corresponding band was observed in non-induced controls (Figure 1B). Upon IPTG induction, cell growth was comparable across induction conditions, although induced cultures exhibited a significant reduction in growth relative to the non-induced control (Figure 1C), consistent with the metabolic burden associated with recombinant protein expression. GFP fluorescence normalized to optical density (GFP/OD) increased over time at all IPTG concentrations, showing both increased fluorescence intensity (Figure 1D) and greater fold induction (Figure 1E) relative to basal expression levels. Furthermore, quantification of Western blot band intensities showed trends consistent with the fluorescence measurements (Figure 1F), supporting the use of GFP fluorescence for monitoring relative FAST-PETase–sfGFP expression. Subsequently, the FAST-PETase–sfGFP reporter system was used to comparatively characterize σ^32^-dependent promoter activity.

### 2.2. Comparative Screening of σ^32^-Dependent Promoters

**Figure 2:**
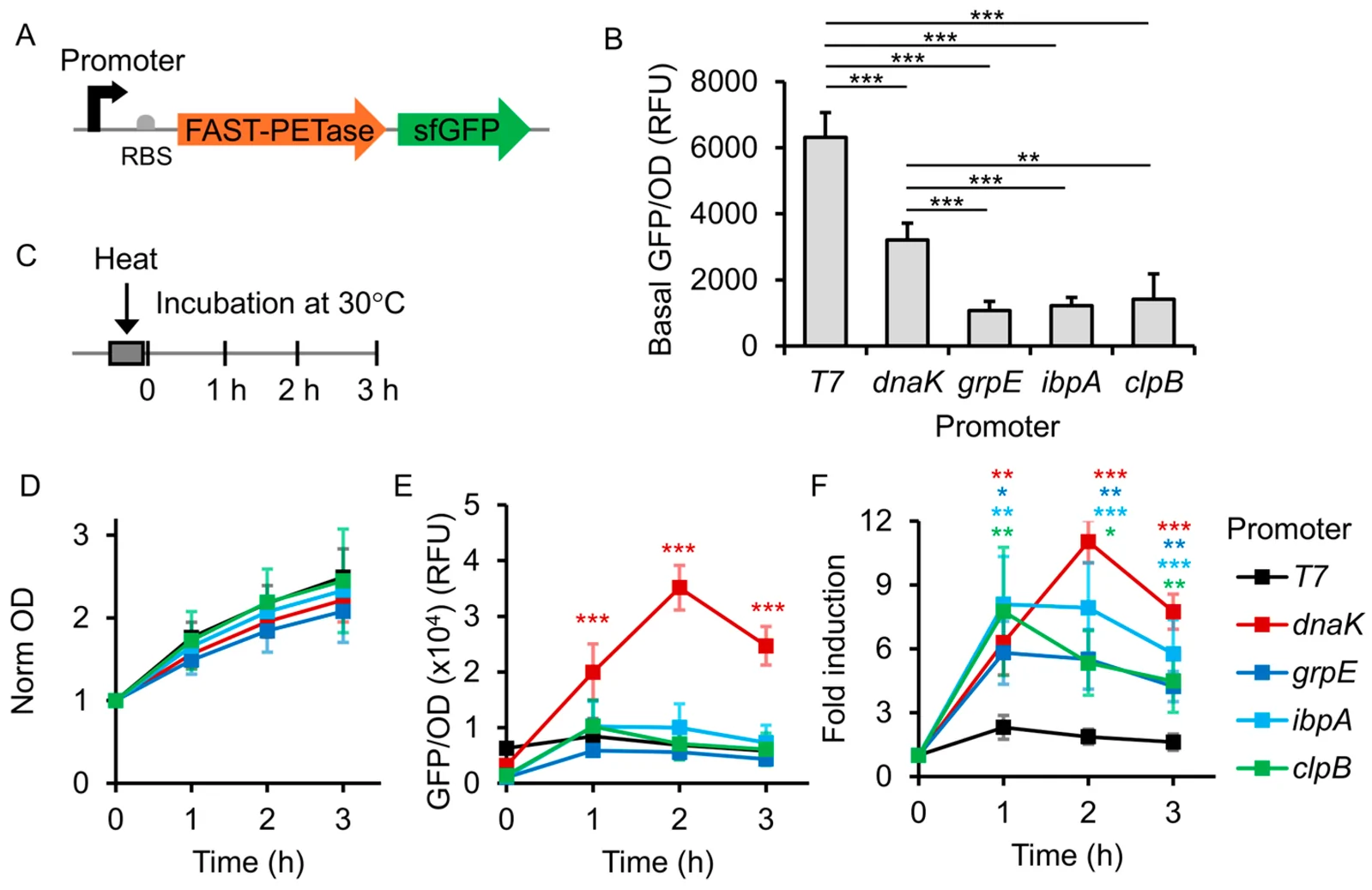
Screening of σ^32^-dependent promoters. (**A**) Schematic of FAST-PETase–sfGFP constructs with T7 or HS promoters. (**B**) Basal GFP/OD levels for each promoter construct prior to heat induction. (**C**) Heat induction workflow. Expression dynamics following heat induction were assessed by (**D**) OD_600_ normalized to the basal value (0 h), (**E**) GFP fluorescence normalized to OD_600_ (GFP/OD, RFU), shown as absolute values to illustrate differences in expression magnitude across promoters, and (**F**) the same data as in (**E**), normalized to the basal value for each promoter and expressed as fold induction relative to basal expression. Data in (**B**,**D**–**F**) are represented as mean ± SD, *n* = 3 independent biological replicates. Statistical significance was assessed by one-way ANOVA followed by Tukey’s HSD post hoc test. For clarity, only comparisons between each HS promoter and the T7 promoter at the corresponding time point are shown. Asterisks indicate significant pairwise differences (* *p* < 0.05, ** *p* < 0.01, and *** *p* < 0.001).

A heat-inducible expression system was constructed by placing σ^32^-regulated HS promoters, including P*dnaK*, P*grpE*, P*ibpA*, and P*clpB*, upstream of FAST-PETase (Figure 2A). Prior to heat induction, the construct with T7 promoter exhibited the highest basal GFP/OD signal, while all HS promoters showed minimal basal leakage (Figure 2B), indicating reduced transcriptional leakage. Upon heat induction at 42 °C (Figure 2C), all constructs displayed comparable growth profiles (Figure 2D). This indicated that promoter selection did not measurably affect cell growth under heat induction. GFP expression also increased over time for all HS promoters, with distinct induction kinetics (Figure 2E,F). P*grpE* displayed both low basal leakage and weak expression. P*ibpA* and P*clpB* showed rapid initial induction, but only P*ibpA* maintained elevated expression throughout the time course. In contrast, P*dnaK* produced the highest expression level with the greatest fold induction among the promoters evaluated while maintaining substantially lower basal expression than the T7 promoter (Figure 2B,F).

Because P*dnaK* consistently exhibited the strongest overall performance, it was selected for characterization of its individual regulatory elements. Additional optimization experiments demonstrated that P*dnaK* promoter activity remained consistent across different induction durations and heat-shock modes at 42 °C, whereas induction at 50 °C impaired both growth and expression (Figure S2). Therefore, a single heat shock at 42 °C for 15 min was used in all subsequent experiments.

### 2.3. Functional Characterization of the PdnaK Promoter

**Figure 3:**
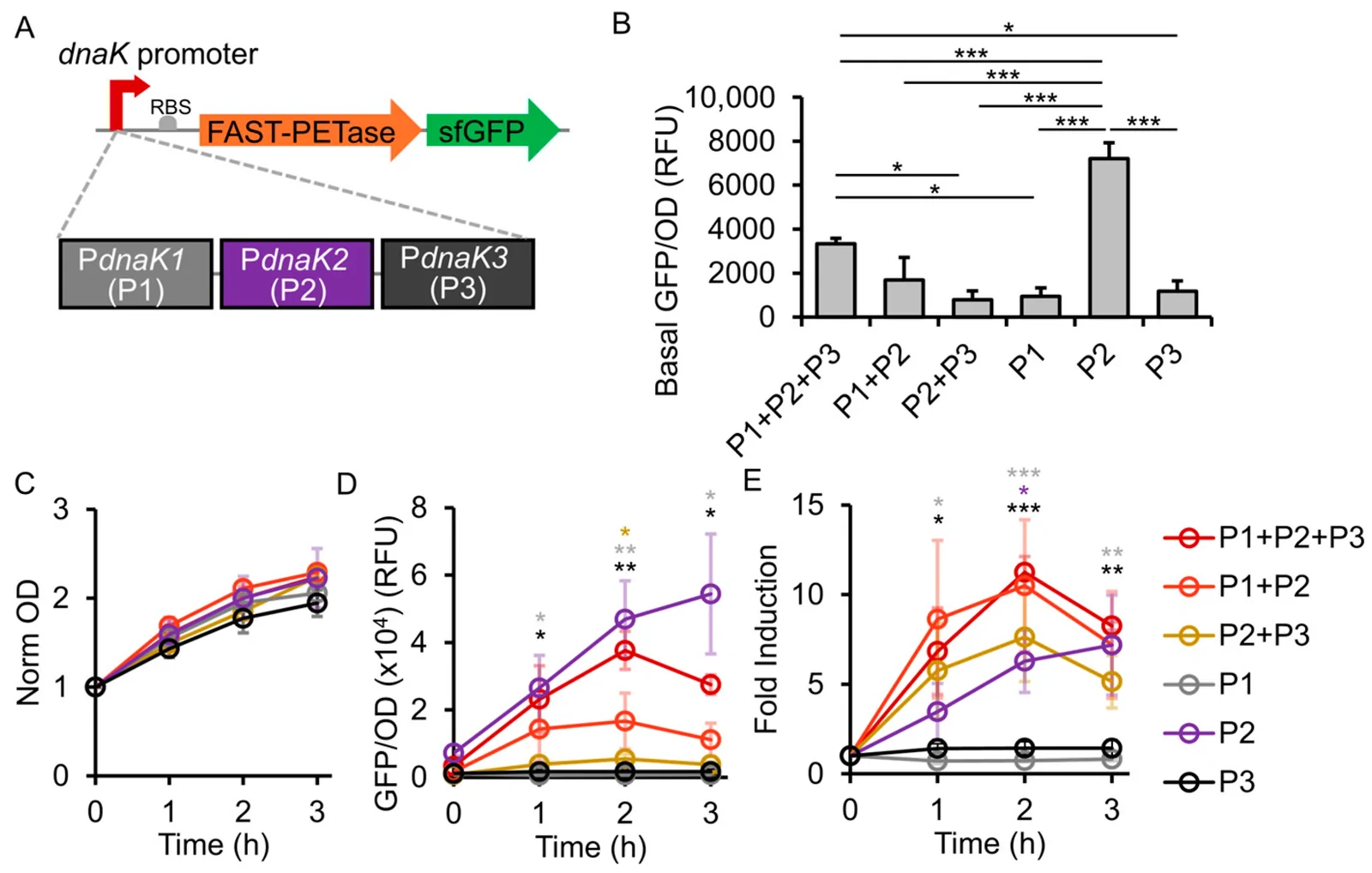
Functional analysis of P*dnaK* promoter elements. (**A**) Schematic of the FAST-PETase-sfGFP construct containing the P*dnaK* promoter, which consists of three elements: P*dnaK1* (P1), P*dnaK2* (P2), and P*dnaK3* (P3). (**B**) Basal GFP/OD levels for different promoter configurations. Expression dynamics following heat induction were assessed by (**C**) OD_600_ normalized to the basal value (0 h), (**D**) GFP fluorescence normalized to OD_600_ (GFP/OD, RFU), shown as absolute values to illustrate differences in expression magnitude across P*dnaK* promoter elements, and (**E**) the same data as in (**D**), normalized to the basal value for each promoter and expressed as fold induction relative to basal expression. Data in (**B**–**E**) are represented as mean ± SD, *n* = 3 independent biological replicates. Statistical significance was assessed by one-way ANOVA followed by Tukey’s HSD post hoc test. Asterisks indicate significant pairwise differences (*p < 0.05, ** *p* < 0.01, and *** *p* < 0.001). For clarity (**C**–**E**), only comparisons between each HS promoter element and the P1 + P2 + P3 element at the corresponding time point are shown.

As P*dnaK* exhibited the strongest performance among the σ^32^-dependent promoters, its regulatory behavior was further investigated. The P*dnaK* promoter region contains three promoter elements: P*dnaK1* (P1), P*dnaK2* (P2), and P*dnaK3* (P3) (Figure 3A). Although these regions have been previously described in studies of the native heat shock response [39,40], their individual sequence features (Figure S3) and contributions to basal leakage and heat-inducible recombinant protein expression have not been systematically evaluated.

Each P*dnaK* element was derived from its native sequence and evaluated individually and in combination with the same synthetic reporter architecture (Figure 3A), allowing their contributions to basal expression (Figure 3B) and heat inducibility (Figure 3C–E) to be compared. Varying the promoter-element composition had no measurable effect on cell growth (Figure 3C). However, differences in reporter expression were observed. Neither P1 nor P3 alone showed detectable heat-inducible expression under the conditions tested. In contrast, P2 alone exhibited the highest basal GFP/OD and the highest absolute GFP/OD at all time points (Figure 3B,D). Incorporation of either P1 or P3 into P2 substantially reduced both basal expression and absolute fluorescence while maintaining heat inducibility. Consequently, all promoter configurations containing P2 exhibited comparable fold induction despite differences in absolute expression (Figure 3E). To further examine promoter dynamics of the P2-containing configurations, expression was monitored for up to 6 h following heat induction. P2 alone continued to increase in expression throughout the extended time course, whereas constructs containing P1 and/or P3 reached maximal expression at approximately 2 h and subsequently showed a gradual decline (Figure S4). These results indicate that the promoter-derived elements contribute differently to the magnitude and temporal profile of heat-inducible expression within the synthetic reporter architecture used in this study.

### 2.4. Structural Interpretation of σ^32^ Interactions with PdnaK Promoter Elements

**Figure 4:**
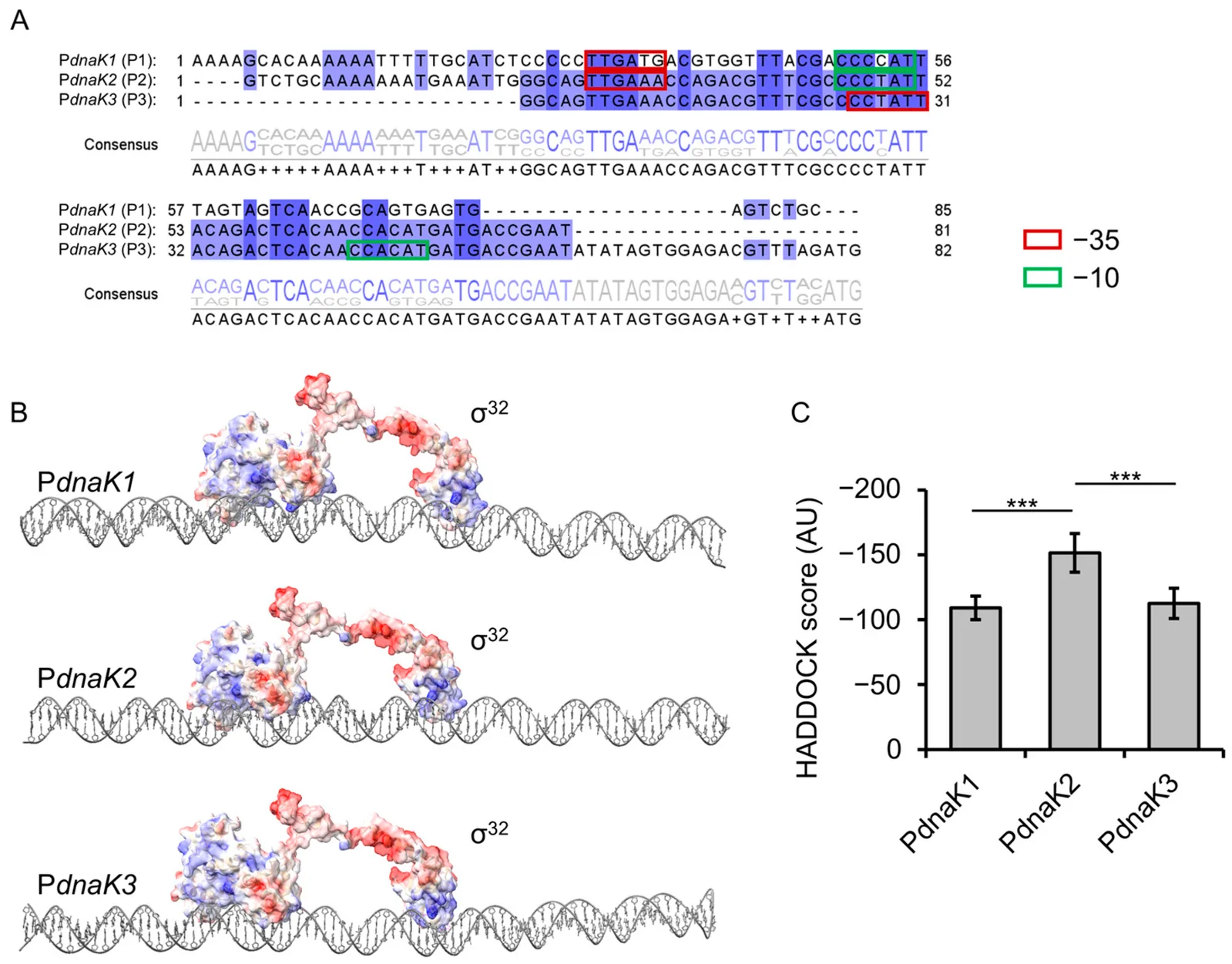
Molecular docking between σ^32^ and *dnaK* promoter elements. (**A**) Multiple sequence alignment of the *dnaK* promoter elements (P*dnaK1*, P*dnaK2*, and P*dnaK3*). The −35 and −10 regions are highlighted in red and green boxes, respectively. The purple color gradient indicates nucleotide percentage identity among the aligned promoter elements, with darker shades representing higher sequence identity. (**B**) Representative docking pose of the σ^32^–DNA complex. σ^32^ is shown as a surface representation colored by electrostatic potential (blue indicates positive charge and red indicates negative charge). The *dnaK* promoter DNA is shown as a double-stranded helix. (**C**) HADDOCK docking scores for interactions between σ^32^ and each *dnaK* promoter element. Data in (**C**) are presented as mean ± SD. Statistical significance was assessed by one-way ANOVA followed by Tukey’s HSD post hoc test. Asterisks indicate significant pairwise differences (*** *p* < 0.001).

Sequence alignment and molecular docking analyses were performed to provide structural context for the experimentally observed activities of the three P*dnaK* promoter-derived elements. Alignment of the conserved −35 and −10 regions showed sequence variation among P1, P2, and P3 that may influence their interactions with σ^32^ (Figure 4A). Molecular docking was then performed to qualitatively compare the predicted σ^32^–DNA interactions of the individual promoter elements. To evaluate the robustness of the molecular docking analysis, two independent σ^32^ structural models and the individual P*dnaK* promoter elements, generated by AlphaFold and SWISS-Model/ChimeraX, were analyzed. Docking calculations were guided by experimentally identified σ^32^ DNA-contact residues T128 and K130 [41] together with additional interface residues (E265, R266, and R268), identified from the modeled complex (PDB ID: 8HKC), against the −35 and −10 regions of each P*dnaK* element. Representative docking poses showed predicted interactions between σ^32^ and each promoter element (Figure 4B).

The docking results showed that the P2 promoter element exhibited the most favorable predicted σ^32^–DNA interaction among the three elements (more negative HADDOCK score), whereas P1 and P3 displayed less favorable docking scores (Figure 4C). This same relative trend was observed with both AlphaFold-derived (Figure 4C) and SWISS-Model/ChimeraX-derived structural models (Figure S5), indicating consistency between the two structural models. Consistent with the experimental promoter characterization, P2 also exhibited the highest basal and heat-induced expression, while P1 and P3 alone showed little or no detectable activity under the conditions tested. Thus, the favorability of the predicted σ^32^–DNA interactions showed qualitative agreement with the observed promoter activities, providing structural context for the functional differences among the P*dnaK* promoter-derived elements.

## 3. Discussion

In this study, four *E. coli* σ^32^-dependent promoters were comparatively characterized to evaluate their performance in heat-inducible recombinant protein expression using FAST-PETase as a model recombinant protein. Although all promoters responded to thermal induction, they displayed distinct basal leakage, induction kinetics, and expression strengths, reflecting differences in their regulatory behavior within the bacterial heat shock response. P*dnaK* exhibited the strongest heat-inducible expression and was selected for detailed functional analysis. Dissection of its promoter element further showed that P*dnaK2* exhibited the strongest autonomous promoter activity. These findings enhance our understanding of the functional behavior of σ^32^-dependent promoters and provide practical information for selecting heat-inducible promoters for recombinant protein expression in *E. coli*.

The distinct expression profiles observed across the σ^32^-dependent promoters reflect their biological functions during the native heat shock response. P*ibpA* and P*clpB* exhibited rapid but transient induction, consistent with their functions in early stress response [42,43,44]. The transient behavior of P*clpB* aligns with previous studies showing a sharp induction peak followed by rapid attenuation, indicating tight temporal regulation of its energy-intensive ATP-dependent activity [45,46]. In contrast, P*dnaK* supported sustained expression following heat induction, consistent with the central role of the DnaK chaperone system in maintaining protein homeostasis throughout the stress response [47]. P*grpE* displayed both minimal basal activity and weak induction, which agrees with its role as a nucleotide exchange factor and thermosensor within the DnaK regulatory network [48]. These findings suggest that the expression behavior of each promoter is closely linked to the native physiological function of the corresponding heat shock gene.

Among the promoters examined, P*dnaK* produced the highest expression level following heat induction, reaching maximal fluorescence approximately 2 h after induction, with maximum absolute sfGFP fluorescence 22.6-fold ± 3.1 higher than basal levels and a corresponding fold induction of 11.2-fold ± 0.9 (*n* = 3). This induction profile is comparable with previous report describing P*dnaK*-regulated recombinant protein expression, which typically reaches maximal activity within 1–4 h after heat shock depending on induction conditions [49,50,51,52]. The subsequent decline in expression beyond the 2 h peak is also consistent with the well-characterized negative feedback regulation of σ^32^ by the DnaK chaperone system. As heat shock proteins accumulate following induction, DnaK reassociates with σ^32^ and promotes its degradation via the FtsH protease, leading to attenuated σ^32^-dependent transcription [33,34]. In addition, reduced translation efficiency may contribute to the reduction in reporter expression [50]. Together, the temporal expression profile of P*dnaK* follows the dynamics of the endogenous heat shock response.

Analysis of the individual P*dnaK* promoter-derived elements, which comprise P1, P2, and P3, provided insight into how promoter composition influences basal activity and inducible expression. Because these elements were evaluated within a synthetic reporter system, their measured activities reflect the autonomous behavior of the corresponding promoter-derived sequences and should not be interpreted as direct measurements of their individual activities within the native P*dnaK* [39]. Systematic testing of the different element combinations showed that P2 exhibited the strongest autonomous promoter activity, whereas P1 and P3 alone showed no detectable expression in this experimental context. Interestingly, incorporation of either P1 or P3 together with P2 significantly reduced basal expression while preserving heat inducibility. These findings suggest that P1 and P3 can modulate the expression behavior of P2. The distinct expression characteristics observed among the σ^32^-dependent promoters provide guidance for selecting promoter configurations according to the desired balance between basal leakage, induction kinetics, and expression strength.

To provide structural context for the experimental observations, molecular docking was performed to compare the predicted interactions between σ^32^ and the individual P*dnaK* promoter-derived elements. The docking analysis showed that P2 exhibited the most favorable predicted σ^32^–DNA interaction, whereas P1 and P3 displayed less favorable HADDOCK docking scores. This trend was qualitatively consistent with the autonomous promoter activities observed in the synthetic reporter system, in which P2 showed the highest expression while P1 and P3 alone showed no detectable activity. However, the docking analysis evaluated individual promoter-derived elements rather than the complete native promoter architecture. Thus, the docking results provide a structural hypothesis for the differential behavior of the P*dnaK* promoter-derived elements. Furthermore, the stronger predicted interaction of P2 was consistent with its higher basal and heat-induced expression, whereas incorporation of P1 and/or P3 reduced basal expression while preserving heat inducibility. One possible explanation is that P1 and P3 modulate the regulatory behavior of P2 under conditions where intracellular σ^32^ is limiting. This interpretation is compatible with the established regulation of intracellular σ^32^ abundance by the DnaK/FtsH pathway [33,34], particularly under non-heat-shock conditions. Although HADDOCK scores provide relative estimates of docking favorability rather than binding affinities, the predicted interaction trends were qualitatively coherent with the experimental promoter activities. The underlying molecular mechanism, however, remains to be determined. Future studies employing *in vitro* transcription assays, DNase I footprinting, and systematic mutagenesis of the −35/−10 spacer regions will directly assess σ^32^ binding and transcriptional dynamics [53,54].

The heat-inducible system offers a potential alternative to chemically induced recombinant protein expression. Compared with IPTG-based systems, thermal induction eliminates the need for chemical inducers and may simplify the induction process. Heat shock also activates the endogenous chaperone system, which could influence the folding and solubility of recombinant proteins and contribute to functional yield [15]. Although a brief heat shock at 42 °C for 15 min was sufficient to activate P*dnaK* while maintaining normal cell growth, further studies are required to compare this system with conventional IPTG-inducible systems in terms of protein yield, solubility, productivity, and scalability. In addition, evaluating σ^32^-dependent promoters with a broader range of recombinant proteins and induction conditions [55,56] will help determine the general applicability of these promoters for heat-inducible recombinant protein expression.

## 4. Materials and Methods

### 4.1. Plasmid Construction and Transformation

The plasmid pET21b(+)-Is-PETase (Addgene plasmid #112202) was used as the template. For the construction of pET21b-FAST-PETase, a codon-optimized FAST-PETase sequence lacking a signaling peptide was used to replace the WT Is-PETase gene in the pET21b vector between the NdeI and XhoI restriction sites using Gibson Assembly purchased from New England Biolabs (NEB, Ipswich, MA, USA; Cat. No. E2611S). For the construction of heat-inducible systems, the T7 promoter region of pET21b-FAST-PETase was replaced with HS promoters, including P*dnaK*, P*grpE*, P*ibpA*, and P*clpB* (Table S1), between the BglII and NdeI sites using Gibson Assembly. To enable monitoring of protein expression, superfolder green fluorescent protein (sfGFP) was fused to the C-terminus of FAST-PETase with P2A linker in all constructs. All plasmids were verified by Sanger Sequencing (Macrogen, Seoul, Republic of Korea).

For promoter-element characterization, P1, P2, and P3 sequences were defined based on the native P*dnaK* promoter architecture annotated in EcoCyc (Figure 4A and Figure S3, and Table S1). Individual promoter elements were placed upstream of the same synthetic RBS (BBa_B0034), spacer sequence, and FAST-PETase–sfGFP reporter cassette to enable comparison of promoter activity. Thus, the individual-element constructs preserved the corresponding promoter sequences but did not reproduce the complete native sequence context of the P*dnaK* promoter. For combinatorial promoter constructs, P1, P2, and P3 were assembled in their native order, with overlapping sequences retained, followed by the same synthetic RBS–spacer–reporter cassette.

### 4.2. Culture and Induction Conditions

All plasmids were transformed into BL21(DE3) (NEB, Cat. No. C2527I) for T7 promoter-driven expression and BL21 (NEB, Cat. No. C2530H) for HS promoter-driven expression by heat shock transformation. Transformants were selected on Luria–Bertani (LB) (BD Biosciences, Franklin Lakes, NJ, USA; Cat. No. 244620) agar plates supplemented with ampicillin (50 µg/mL). In each induction experiment, at least three colonies were selected as independent biological replicates and subjected to parallel induction experiments. For the IPTG induction, a single colony containing a plasmid with a T7 promoter was inoculated in 5 mL LB medium containing ampicillin and cultured at 30 °C with shaking at 220 rpm for 15 h. The overnight culture was then diluted into 1 mL fresh LB medium containing ampicillin in a 14 mL tube. IPTG (Gold Biotechnology, St. Louis, MO, USA; Cat. No. I2481) was added at the indicated concentrations, and cultures were incubated at 30 °C for 1, 2, or 3 h. For heat induction, a single colony containing a plasmid with an HS promoter was cultured under the same conditions. Cells were subjected to a transient heat shock at 42 °C for 15 min, followed by dilution into 1 mL fresh LB medium containing ampicillin and incubation at 30 °C for 1, 2, and 3 h, unless otherwise specified.

### 4.3. Optical Density and Fluorescence Measurements

Cell cultures were transferred to a 96-well clear-bottom plate (NEST Biotechnology Co., Ltd., Wuxi, China; Cat. No. 701401). Cell growth was monitored by measuring optical density at 600 nm (OD_600_) using a plate reader (Tecan Infinite M Plex, Tecan, Männedorf, Switzerland). Normalized OD (Norm OD) was calculated by dividing OD_600_ at each time point by the value at time 0, reflecting fold change in cell growth. sfGFP fluorescence was measured on the same instrument at excitation and emission wavelengths of 480 nm and 510 nm, respectively, and expressed in relative fluorescence units (RFU). Fluorescence was normalized to cell density (OD_600_) at each time point to yield GFP/OD (RFU) that allows direct comparison of expression magnitude across conditions or promoters. To quantify the increase in expression following heat induction, fold induction was introduced, which was calculated by normalizing GFP/OD value relative to its baseline value at 0 h.

### 4.4. Protein Purification

The pET21b-FAST-PETase plasmid was transformed into BL21(DE3). A single colony was inoculated into 5 mL of LB medium supplemented with 50 µg/mL ampicillin and cultured overnight at 37 °C with shaking at 225 rpm. The overnight culture was diluted (1:10) into 50 mL fresh medium and cultured until the OD_600_ reached 0.6–0.8. Protein expression was induced by the addition of 0.5 mM of IPTG, and cells were further cultured for 24 h at 30 °C, 225 rpm. The induced cell culture was harvested by centrifugation at 4000× *g* at 4 °C for 20 min. Cell pellets were washed with 10 mL Dulbecco’s Phosphate-Buffered Saline (DPBS, Corning, NY, USA; Cat. No. 21-031-CM) and extracted using Dr. Gold’s Bacterial Protein Extraction Reagent (Gold Biotechnology, St. Louis, MO, USA, Cat. No. GB-500), following manufacturer’s instructions. The cells were subsequently lysed by sonication, and the lysate was centrifuged at 14,000 rpm at 4 °C for 30 min to collect the supernatant that contained soluble proteins. Soluble proteins were purified using Ni-NTA affinity chromatography (QIAGEN, Hilden, Germany; Cat. No. 30210) with Bio-Rad columns (Bio-Rad Laboratories, Hercules, CA, USA; Cat. No. 7321010). The eluted proteins were further concentrated using Amicon Ultra centrifugal filters (MilliporeSigma, Burlington, MA, USA; Cat. No. UFC8030) and maintained in DPBS.

Protein concentration was determined using a Qubit 4 Fluorometer (Thermo Fisher Scientific, Waltham, MA, USA) with the Qubit Protein Assay Kit, according to the manufacturer’s instructions. The purity of FAST-PETase was estimated by SDS-PAGE followed by densitometric analysis in ImageJ version 1.54d, which was calculated as the intensity of the FAST-PETase band relative to the total band intensity in the lane. The FAST-PETase band represented 13.2% ± 1.1% of total protein (mean ± SD, *n* = 3 independent purifications). Based on this estimate, 7.1 µg of FAST-PETase was used in the PET degradation assay (Figure S1).

### 4.5. SDS-PAGE and Western Blot Analysis

Whole cell lysates and soluble protein samples were analyzed by sodium dodecyl sulfate polyacrylamide gel electrophoresis (SDS-PAGE) using 12% separating gel and 4% stacking gel. Proteins were visualized by Coomassie Brilliant Blue R-250 staining (PanReac AppliChem, Darmstadt, Germany; Cat. No. 42660) and imaged using an Alliance Q9 Advanced imaging system (Uvitec, Cambridge, UK). For Western blot analysis, purified proteins were separated by SDS-PAGE, transferred onto membranes, and probed with an anti-His tag antibody (Thermo Fisher Scientific, Cat. No. MA1-21315-HRP) to detect FAST-PETase. Membranes were imaged using an Alliance Q9 Advanced imaging system (Uvitec, UK).

### 4.6. Evaluation of PET Degradation 

Commercial PET film (6 mm diameter, 1.27 ± 0.08 mg) was incubated with purified FAST-PETase in 100 mM KH_2_PO_4_–NaOH buffer at 50 °C for 96 h. After incubation, PET samples were sequentially washed with 1% SDS, deionized water, and 100% ethanol. To characterize the chemical functional groups of the PET surface, Fourier-transform infrared (FT-IR) spectroscopy (Nicolet iS5, Thermo Scientific, Waltham, MA, USA) was performed on both enzyme-treated and untreated PET samples in attenuated total reflectance (ATR) mode using a diamond crystal. Spectra were recorded over 4000–400 cm^−1^ at a resolution of 4 cm^−1^, averaging 32 scans per sample. The carbonyl index was calculated as the ratio of absorbance at 1713 cm^−1^ (C=O ester stretching) to 1410 cm^−1^. Surface morphology of PET samples was further examined using scanning electron microscopy (SEM; HITACHI TM3030Plus, Tokyo, Japan).

### 4.7. Molecular Docking Analysis

Molecular docking was performed to investigate interactions between σ^32^ and HS promoters using the HADDOCK 2.4 web server [57,58]. Two independent structural models of σ^32^ and P*dnaK* were used. The first model was predicted using AlphaFold3 (AlphaFold Server). The three-dimensional structure of σ^32^ was from the *E. coli* σ^32^ amino acid sequence obtained from the UniProt database (P0AGB3), and the double-stranded DNA (dsDNA) models for each P*dnaK* promoter element were predicted from the nucleotide sequences of each element. Another model of σ^32^ was generated by homology modeling using SWISS-MODEL, based on the 2.49 Å cryo-EM structure of σ^32^ (PDB ID: 8HKC, chain G, [41]) to compensate for unresolved regions in the experimental structure. Model quality was further assessed using SWISS-MODEL’s built-in metrics, yielding a GMQE score of 0.86 and a QMEANDisCo Global score of 0.83 ± 0.05, indicating high model quality and strong agreement with experimentally determined structures [59]. Double-stranded DNA models of the P*dnaK1*, P*dnaK2*, and P*dnaK3* were generated using ChimeraX version 1.11, which constructs idealized B-form dsDNA from input sequence [60].

For both models, docking in HADDOCK was guided by specific σ^32^ residues, including T128 and K130, that have been experimentally identified as σ^32^–DNA contacts [41], together with additional interface residues (E265, R266, and R268) identified from the cryo-EM complex. Thus, docking simulations were performed by defining active residues on σ^32^ (T128, K130, E265, R266, and R268) and the corresponding −35 and −10 promoter regions of each DNA element (Figure 4A). The cluster with the lowest HADDOCK score, the largest cluster size, and the most negative Z-score was selected as the representative binding model and visualized using ChimeraX.

### 4.8. Statistical Analysis

All experiments were performed with at least three independent biological replicates. For time-course experiments, GFP fluorescence was normalized to OD_600_ (GFP/OD) and further normalized to the pre-induction value at 0 h to calculate fold induction. Data are presented as mean ± standard deviation (SD), unless otherwise indicated. Statistical significance was assessed by one-way ANOVA followed by Tukey’s HSD post hoc test for pairwise comparisons, performed in R (version 4.5.1). Differences were considered statistically significant at *p* < 0.05. The specific reference group used for pairwise comparisons is indicated in each figure legend.

## 5. Conclusions

By systematically comparing four native σ^32^-dependent promoters, this study identified P*dnaK* as the highest-performing promoter for heat-inducible FAST-PETase expression in *E. coli*. Dissection of the P*dnaK* promoter-derived elements further showed that P*dnaK2* exhibited the strongest autonomous promoter activity in the synthetic reporter architecture, whereas P*dnaK1* and P*dnaK3* modulated basal expression, consistent with qualitative differences in predicted σ^32^–DNA interactions. These findings suggest that promoter architecture may serve as a useful design parameter for balancing basal leakage and inducible expression in heat-responsive systems. Beyond FAST-PETase, the insights gained from this work may facilitate the engineering of heat-inducible promoters for recombinant enzyme expression in *E. coli*.

## Data Availability

The original contributions presented in this study are included in the article/Supplementary Materials. Further inquiries can be directed to the corresponding author(s).

## Supplementary Materials

The following supporting information can be downloaded at: https://www.mdpi.com/article/10.3390/ijms27177762/s1. References [61,62] are cited in the supplementary materials.
