# Supplementary material for: Comparative Characterization of σ32-Dependent Promoters for the Heat-Inducible Expression of FAST-PETase in Escherichia coli
Source: Int J Mol Sci. 2026 Aug 30;27(17):7762. doi: 10.3390/ijms27177762 (PMC13566873; doi:10.3390/ijms27177762)
Supplement: Supplementary file 1 [file ijms-27-07762-s001.zip › ijms-4505387-supplementary.pdf]

Supplementary Material

Comparative Characterization of σ^{32}-Dependent Promoters for the Heat-Inducible Expression of FAST-PETase in *Escherichia coli*

Praopim Limsakul [1,2], Natcha Rasitanon [3], Sahban Da-oh [4], Amornrat Phongdara [5], Aekkaraj Nualla-ong [4,5,*] and Krit Charupanit [3,*]

1 Division of Physical Science, Faculty of Science, Prince of Songkla University, Songkhla 90110, Thailand; praopim.l@psu.ac.th
2 Center of Excellence for Trace Analysis and Biosensor (TAB-CoE), Prince of Songkla University, Songkhla 90110, Thailand
3 Department of Biomedical Sciences and Biomedical Engineering, Faculty of Medicine, Prince of Songkla University, Songkhla 90110, Thailand; 6810330024@psu.ac.th
4 Division of Biological Science, Faculty of Science, Prince of Songkla University, Songkhla 90110, Thailand; sahban0774@gmail.com
5 Center for Genomics and Bioinformatics Research, Faculty of Science, Prince of Songkla University, Songkhla 90110, Thailand; pamornra@yahoo.com
* Correspondence: aekkaraj.n@psu.ac.th (A.N.-o.); krit.ch@psu.ac.th (K.C.)

FAST-PETase catalytic validation

To verify that the codon-optimized enzyme retained its catalytic function, FAST-PETase was expressed from an IPTG-inducible T7 promoter (Fig. S1A). Purified FAST-PETase was incubated with commercial PET films at 50 °C for 96 h, and its degradation was evaluated by FTIR spectroscopy, weight-loss analysis, and scanning electron microscopy (SEM). FTIR analysis revealed reduced absorbance at 1713 cm^{-1}, which corresponds to the ester carbonyl stretching vibration of PET, following enzyme treatment (Fig. S1B). The carbonyl index, calculated as the absorbance ratio at 1713 to 1410 cm^{-1}, was significantly decreased compared with untreated controls (Fig. S1C), indicating hydrolysis of the PET polymer backbone. Enzyme-treated PET films also showed about 6% weight loss (Fig. S1D). Consistently, SEM analysis (Fig. S1E-F) demonstrated surface erosion (Fig. S1F) compared with the smooth surfaces of untreated PET films (Fig. S1E). These results confirmed that the codon-optimized FAST-PETase retained catalytic activity and was suitable for use as the model recombinant protein in this study.

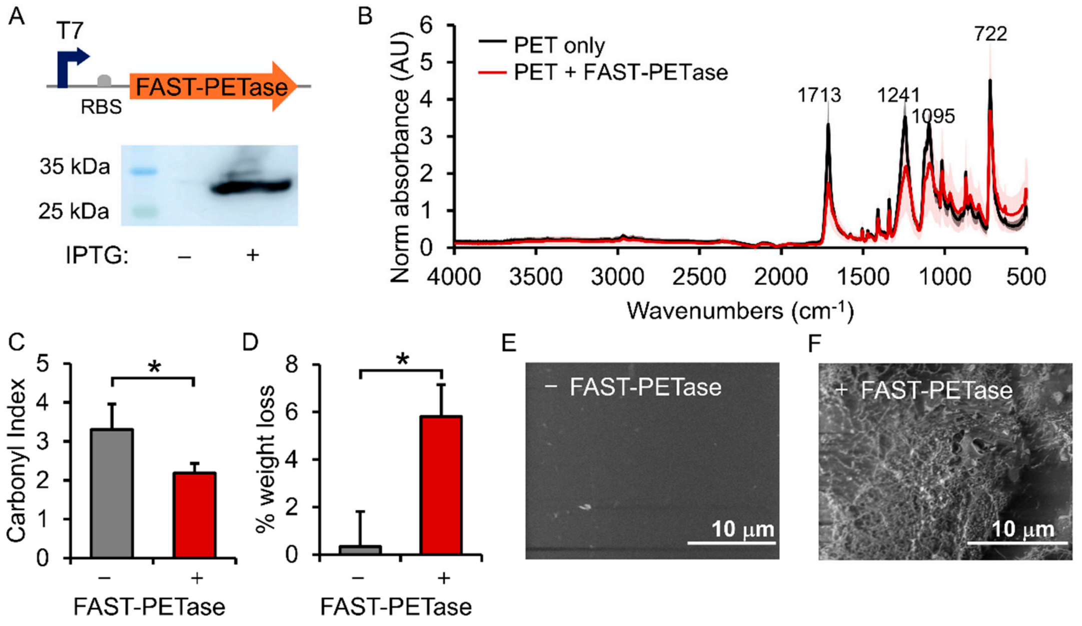

Figure S1. Expression and catalytic activity of FAST-PETase.

(A) Schematic of FAST-PETase constructs driven by T7 promoter (top) and Western blot analysis (bottom) of its expression under non-induced (− IPTG) and induced (+ IPTG) conditions. The expected molecular weight of FAST-PETase is 27.9 kDa. (B) FTIR spectra of PET substrates following incubation with (red) or without (black) FAST-PETase. Solid lines represent the mean, and shaded areas represent SD. (C) The carbonyl index (ratio of absorbance at 1713 cm^{-1} to 1410 cm^{-1}), and (D) % weight loss analysis of PET substrates incubated without (−) and with (+) FAST-PETase. (E, F) Representative SEM images of PET surfaces (E) without enzyme treatment and (F) after incubation with FAST-PETase. Data in (C-D) are represented as mean ± SEM, n = 3 independent biological replicates. Statistical significance was assessed by Student's t-test. Asterisks indicate significant pairwise differences (*p<0.05).

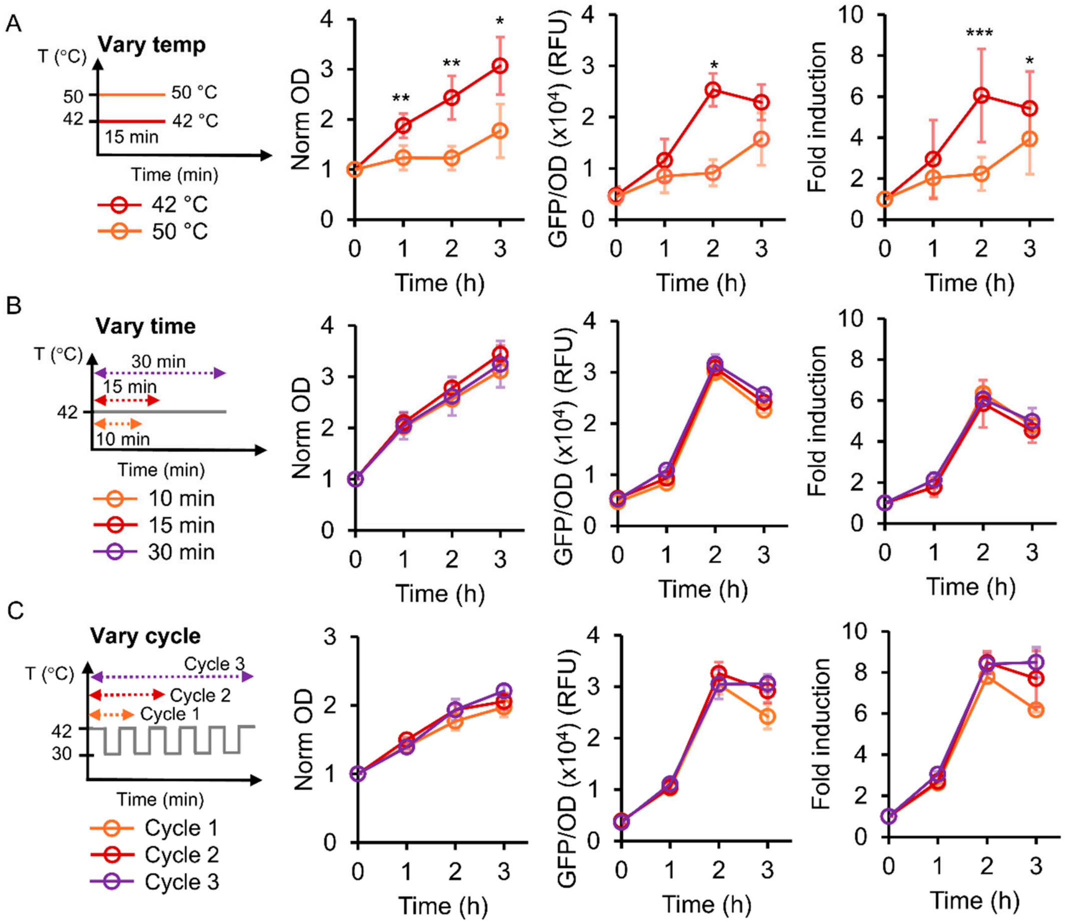

Figure S2. Optimization of heat induction parameters for P*dnaK*-driven expression.

Effect of heat induction parameters, including (A) temperature, (B) heat treatment duration, and (C) cyclic heat treatment, on FAST-PETase–sfGFP expression. For each condition, expression dynamics were evaluated using OD_{600} normalized to the pre-induction (0 h) value (left), GFP fluorescence normalized to OD_{600} (GFP/OD, RFU; middle), and fold induction relative to basal levels (right). Data in (A-C) are represented as mean ± SD, $n = 5$ (A), $n = 3$ (B), and $n = 3$ (C) independent biological replicates. Statistical significance was assessed by Student's t-test. Asterisks indicate significant pairwise differences (*p<0.05, ** p<0.01, and *** p<0.001).

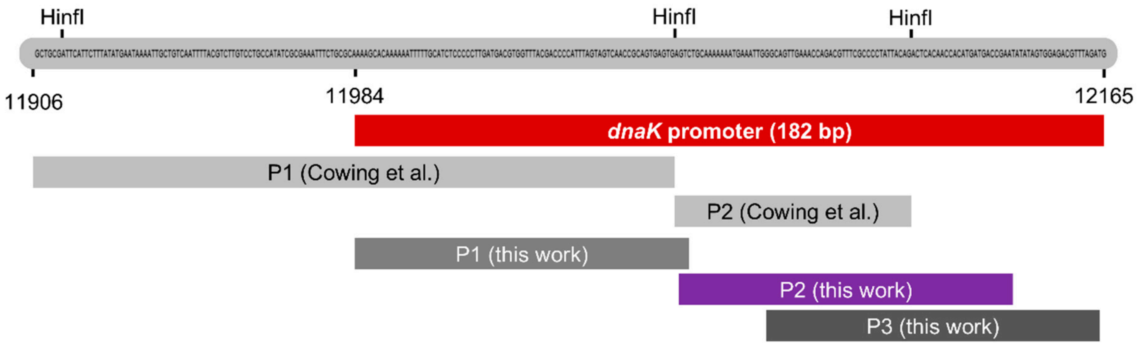

Figure S3. Sequence organization of the *dnaK* promoter and promoter fragments analyzed in Cowing et al. [39] and this study.

The 260-bp genomic region of *Escherichia coli* K-12 substr. MG1655 (GenBank accession NC_000913.3, positions 11,906–12,165) encompassing the *dnaK* promoter is shown. The 182-bp *dnaK* promoter sequence used in this study corresponds to the native *dnaK* promoter sequence characterized by Cowing et al. The P1 and P2 fragments analyzed by Cowing et al. and the P1, P2, and P3 promoter elements analyzed in this study are indicated for comparison.

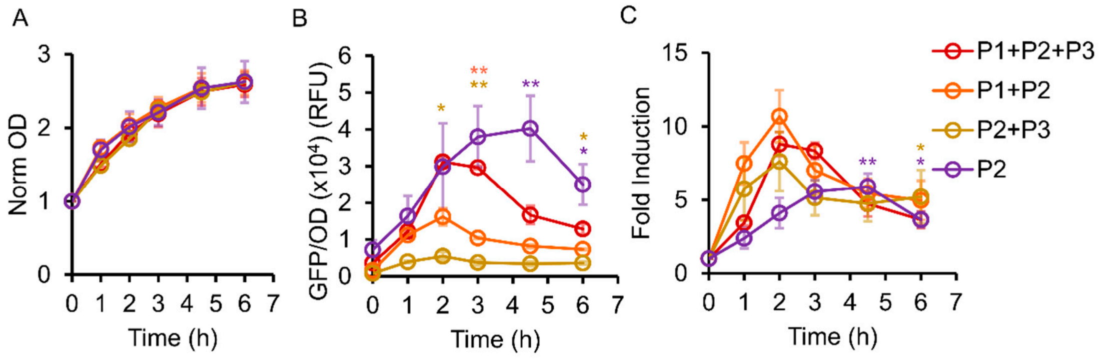

Figure S4. Extended time-course FAST-PETase expression under the *dnaK* promoter elements.

Expression dynamics following heat induction were assessed by (A) OD_{600} normalized to the basal value (0 h), (B) GFP fluorescence normalized to OD_{600} (GFP/OD, RFU), shown as absolute values to illustrate differences in expression magnitude across *dnaK* promoter elements, and (C) the same data as in (B), normalized to the basal value for each promoter and expressed as fold induction relative to basal expression. Data are represented as mean ± SD, $n = 3$ independent biological replicates. Statistical significance was assessed by one-way ANOVA followed by Tukey's HSD post-hoc test. For clarity, only comparisons between each HS promoter element and the P1+P2+P3 element at the corresponding time point are shown. Asterisks indicate significant pairwise differences (*p<0.05, **p<0.01).

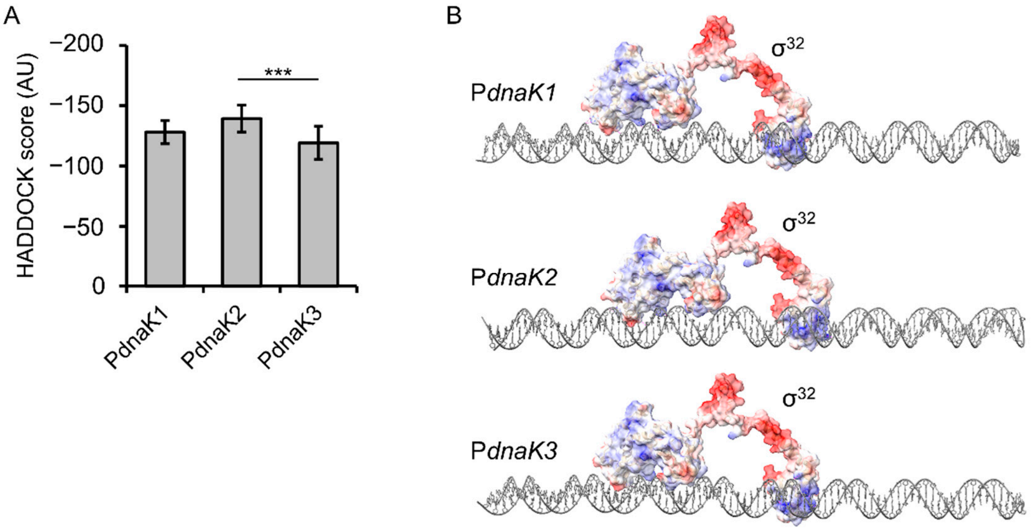

Figure S5. Molecular docking between σ^{32} and *dnaK* promoter elements using the SWISS-MODEL/ChimeraX-predicted structures.

(A) HADDOCK docking scores for interactions between σ^{32} and each *dnaK* promoter element. Data in (A) are presented as mean ± SD. Statistical significance was assessed by one-way ANOVA followed by Tukey's HSD post-hoc test. Asterisks indicate significant pairwise differences (****p*<0.001). (B) Representative docking pose of the σ^{32}–DNA complex. σ^{32} is shown as a surface representation colored by electrostatic potential (blue indicates positive charge and red indicates negative charge). The *dnaK* promoter DNA is shown as a double-stranded helix.

Table S1. Nucleotide sequence of σ^{32}-dependent promoters [61,62].

Promoter	Sequence
P*dnaK*	AAAAGCACAAAAAATTTTTGCATCTCCCCCTTGATGACGTGGTTTACGACCCCATTTAGT AGTCAACCGCAGTGAGTGAGTCTGCAAAAAAATGAAATTGGGCAGTTGAAACCAGACG TTTCGCCCCTATTACAGACTCACAACCACATGATGACCGAATATATAGTGGAGACGTTTA GATG
P*grpE*	CCGAGGTCCTTGTTGCGAAGATTGATGACAATGTGAGTGCTTCCCTTGAAACCCTGAAA CTGATCCCCATAATAAGCGAAGTTAGCGAGATGAATGCG
P*ibpA*	TGCAAAAAAAAGTCCGCTGATAAGGCTTGAAAAGTTCATTTCCAGACCCATTTTTACAT CGTAGCCGATGAGGACGCGCCT
P*clpB*	TTTTCACATTAATCTGGTCAATAACCTTGAATAATTGAGGGATGACCTCATTTAATCTCC AGTAGCAACTTTGATCCGTTA

Table S2. HADDOCK docking results obtained using the AlphaFold3-predicted structures.

Binding complex between σ^{32} and	HADDOCK Score	Z-Score	Buried Surface Area (A°2)	Van der Waals Energy (kcal/mol)	Electrostatic Energy (kcal/mol)
P*dnaK1*	-109.1 ± 9.1	-1.2	1608.6 ± 66.6	-23.3 ± 3.3	-586.8 ± 30.5
P*dnaK2*	-151.6 ± 14.9	-2.0	2201.8 ± 249.6	-36.5 ± 5.3	-736.7 ± 54.7
P*dnaK3*	-112.6 ± 11.6	-0.6	1843.9 ± 216.0	-25.1 ± 7.1	-598.2 ± 36.6

Table S3. HADDOCK docking results obtained using the SWISS-MODEL/ChimeraX-predicted structures.

Binding complex between σ^{32} and	HADDOCK Score	Z-Score	Buried Surface Area (A°2)	Van der Waals Energy (kcal/mol)	Electrostatic Energy (kcal/mol)
P*dnaK1*	-128.1 ± 9.6	-1.9	2110.1 ± 93.1	-27.7 ± 5.3	-680.9 ± 58.4
P*dnaK2*	-139.3 ± 11.2	-1.9	2342.7 ± 165.5	-42.9 ± 9.3	-635.3 ± 10.9
P*dnaK3*	-119.2 ± 13.7	-1.7	2014.6 ± 199.0	-42.8 ± 6.3	-539.9 ± 61.7
